# Patient Opinions About Foreign Body Contraceptives

**DOI:** 10.1089/whr.2020.0048

**Published:** 2020-10-08

**Authors:** Grace P. Ferguson, Tiffany Deihl, Kimberly Bell, Judy C. Chang

**Affiliations:** Department of Obstetrics, Gynecology and Reproductive Sciences, University of Pittsburgh Medical Center, Pittsburgh, Pennsylvania, USA.

**Keywords:** contraception, foreign body, implant, intrauterine device, patient attitude, patient perspective, “up in me”

## Abstract

***Background:*** Prior studies have noted patient reluctance to use contraceptive devices that require insertion into their bodies. We sought to better understand this “foreign body” concern, as well as to clarify how women perceive long-acting reversible contraception (LARC) devices compared with other implanted medical devices.

***Materials and Methods:*** We performed semistructured qualitative individual interviews with female obstetric/gynecologic patients and probed their opinions regarding LARC devices. Trained coders analyzed interview content using an inductive iterative approach and identified key themes.

***Results:*** We found three major themes in our analysis. First, women frequently expressed uncertainty about where in the body intrauterine devices reside and the impact of a foreign body in that space. Second, women expressed discomfort with the invisibility of the device itself and the “set and forget” feature of LARCs. Finally, when asked to consider contraceptive devices in the context of other implantable medical devices, patients highlighted that contraceptive devices are elective and have alternative options.

***Conclusions:*** When women express concerns about contraceptive devices “up in them,” they are expressing concerns about how these devices interact with their anatomy and the possibilities of harm and failure. These perceived risks of LARCs may not compare favorably with other contraceptive methods that are not foreign bodies. Understanding this perspective improves our ability to participate in shared decision-making.

## Introduction

Long-acting reversibl contraceptives (LARCs) are a highly effective and safe method of contraception.^1^ From 2008 to 2014, LARC use in contracepting women in the United States increased from 6% to 14%, made up primarily of women switching from moderately effective methods to LARC.^2^ As LARC usage has increased, so has research centered on both incentives and disincentives related to LARC devices. Some incentives noted in the literature include convenience, efficacy, long duration, and less hormones. Disincentives include fear of side effects, potential effects on future fertility, long duration, fear of insertion, barriers to medical access, cost, and unease with a foreign body.^3–5^ The first author was prompted to conduct the present research due to personal clinical experience with patients expressing concerns about contraception as a foreign body.

The “foreign object” objection to LARC devices has been frequently demonstrated in the literature.^5–9^ Kavanaugh et al. performed structured interviews with 48 patients to explore their perspectives on LARCs and found that the most common disadvantage mentioned was that the device is a “foreign object.” Notably, although this was a very common theme among the interviewed patents, this concept was not cited by any providers.^10^ Another set of interviews regarding intrauterine device (IUD) perspectives demonstrated that 22 out of 40 women stated that they did not want something “up in me.”^11^ Two other qualitative studies focusing on LARC perspectives identified common negative themes of “something in the uterus unappealing”^12^ and a “foreign body” as an infection risk.^13^ One study even characterized this discomfort with a foreign body as, “Oh, it's an alien,” with women sharing that LARC methods were “scary” and “creepy.”^3^ It is evident that the “up in me” concern predominantly refers to IUDs in previous research; however, the “foreign body” concept has been cited with respect to both IUDs and implants.^5,6^

To our knowledge, no studies have specifically addressed patients' underlying concerns regarding the perceived disadvantage of an LARC as a “foreign body.” In addition, much of the previous literature has focused on the perspectives of adolescent and college-aged women. Therefore, we sought to investigate a broad age range of patients' concerns specifically related to the concept of an LARC as a foreign body, specifically what is meant when someone says, “I don't want that up in me,” and to explore patient-perceived differences between LARCs and other implanted medical devices.

## Materials and Methods

This was a qualitative study that utilized semistructured individual patient interviews, which allowed women to describe their experiences using their own words.^14^ We chose to use individual interviews as we felt that the privacy would allow women more freedom to express their thoughts regarding this abstract and private subject.^15^ Prior research has indicated that thematic saturation for in-depth interviews occurs between 12 and 25 interviews, and therefore, we initially targeted to recruit at least 25 participants, with the possibility to expand recruitment should thematic saturation have not been reached at that time.^16,17^

Our study was conducted in the ambulatory gynecologic clinics of Magee-Womens Hospital, a university-affiliated medical center in Pittsburgh, PA, and was approved by the Institutional Review Board of the University of Pittsburgh. There are over 26,000 outpatient visits annually for gynecologic care, and about 90% of patients have government-funded health insurance. English-speaking women aged 14–55 years who presented for care at our outpatient obstetrics and gynecology clinic were eligible for study participation. The study interviewers (G.P.F. and K.B.) approached women in the communal clinic waiting room to invite potential participants to learn more about the study. While both interviewers were also clinicians in the study setting, neither of them were working in that capacity at the time they recruited for the study and were dressed in plain clothes to avoid a coercive influence on participation. We chose not to limit study participation based on prior or current contraceptive choices, as we felt that opinions regarding implanted devices were likely to be found in women both with and without a personal history of LARC use. Those who expressed interest were escorted to an adjacent private space designated for research, where they were provided details about the study topic, objectives, format, and an opportunity to decide on participation. Women who expressed interest in participating in the study were then verbally consented as approved by the University of Pittsburgh Institutional Review Board.

Given our reluctance to impose any of our own interpretation or biases to this work, we did not approach the design of the interview guide or our analysis with any overriding theoretical or conceptual framework or system. Our intent was to thus perform qualitative description, to describe the perspectives of the women participating in our study in everyday language.^18^ The interview guide focused on open-ended questions to allow the participants to guide the discussion and was refined over the first few interviews. We began by asking participants to describe their familiarity with and understanding of if they were familiar with the contraceptive implant or IUDs, what they knew about them, and if they had opinions or experiences with any of these devices. If the participant did not spontaneously bring up the theme “I don't want *that* up in me,” or a similar foreign body statement, we prompted this discussion by stating: “Some women have… What do you think they might mean by that?” The final part of the interview focused on the perceived differences between LARC devices and other implanted medical devices. In this part of the interview, we asked participants to compare LARC devices with medical devices such as a pacemaker, insulin pump, organ transplant, or breast implant. The diversity of objects chosen for comparison was meant to illustrate a wide variety of indications for use. After the interview, participants filled out a brief demographic survey, including information about insurance status, number of health visits, and level of education, but not including current or previous contraceptive use. Participants were also provided with a factsheet on LARC devices, and were provided a monetary compensation of $20 in the form of a cash card.

Interviews were conducted by the PI (G.P.F.) and another investigator (K.B.), who were both trained by an experienced qualitative interviewer (J.C.C.) with practice interviews and regular debriefing. Interviews were audio-recorded, professionally transcribed, and loaded for storage and organization into ATLAS.ti software.

Our analytic approach was thematic analysis as our intent was to provide meaning and understanding; not to simply describe—we sought an interpretative level that helped foster understanding of how the perspectives and views described by our participants offered insight to women's reticence in considering foreign body contraceptives.^19^ We used an open and inductive approach for data analysis. Two coders (G.P.F. and T.D.) independently reviewed all interview transcripts and assigned interpretative codes. Each coder initially used memoing as a tool to note patterns and categories observed from the data. These memos allowed coders to refine and adapt the codebook to more deeply explore some of these trends. Throughout analysis, the coders met to compare and discuss their coding. We assigned a third investigator (J.C.C.) to adjudicate coding disagreements, however, no such disagreements occurred. The final codebook was reapplied to all transcripts.

When all transcripts had been coded using the final codebook, G.P.F. and T.D. reviewed the codes to identify categories and patterns. Relationships between codes and categories allowed us to identify themes and subthemes. These were reviewed with the full study team, and in these discussions, we considered how the themes related to one another and what type of meaning was contained within and across them. Wishing to stay close to the women's words and narratives, we chose a semantic approach to our thematic analysis and thus frame our themes within the content of our interviews.^19,20^

## Results

### Participant characteristics

Twenty-five cis-gendered women participated in this qualitative study. Thematic saturation was noted at the 20th interview, but as our subject recruitment and data collection had outpaced the analysis that noted occurrence of saturation, 25 total interviews were conducted, and all included in the analysis. The median age was 26 (16–49), and 72% of our participants identified as African American. Most participants had graduated high school or completed some college. More than half the women were multiparous. Participant characteristics are provided to contextualize our analysis in Table 1. Participant quotes are presented with pseudonyms to protect anonymity but give context, as well as demographic information about self-reported race and age.

**Table 1.** Participant Demographics

Characteristics	n = 25
Age	
16–20	6
21–25	7
26–30	8
30+	4
Race	
White	4
Black	18
Mixed	3
Ethnicity	
Hispanic	1
Not Hispanic or Latina	17
Unspecified	7
Education	
Some high school	2
Graduated high school	8
Some college	11
Graduated college	2
Professional school	1
Unspecified	1
Gravidity	
0	4
1	4
2	4
3+	12
Unspecified	1
Living children	
0	5
1	8
2	5
3+	6
Unspecified	1
Prior abortion?	
Yes	7
No	12
Unspecified	6
Clinic visits (past year)	
1	4
2	5
3–4	4
5+	8
Unspecified	4

### Themes

We found three major themes in our analysis.

First, women frequently expressed uncertainty about where in the body intrauterine devices reside and the impact of a foreign body in that space. Second, women expressed discomfort with the invisibility of the device itself, which made them question its safety and efficacy. Finally, when asked to consider contraceptive devices in the context of other implantable medical devices, patients highlighted that contraceptive devices are elective.

#### Uncertainty regarding location and position

When we discussed the quote “I don't want that up in me” with participants, they overwhelmingly guided the conversation to IUDs. Concern regarding positioning of the IUD within the body was among the most mentioned themes. Their understanding of the location of IUDs within their own body was highly variable. Anatomically, the words used to describe location were varied, with “cervix,” “vagina,” “ovaries,” fallopian tubes,” and “uterus” all being used interchangeably. The language being used to describe anatomy was inexact, illustrating a possible breakdown in patient/provider communication.

Conceptually, the positioning of an IUD was a major point of concern for participants. Many women expressed concern that the device would or could be free floating within their bodies. One participant stated that the IUD was “…sort of floating around your fallopian tubes” (Aliyah, Black, 24) and another that, “It could probably slip into your blood cells … get lost in the blood stream” (Alexis, Black, 19). One woman simply replied, “There are a lot of places it can get lost [laugh]” (Kayla, Black, 26). Women also worried about device movement in and out of the body, stating: “I think the fear of it being lodged in there or the fear of it coming out and not knowing” (Jennifer, White, 49). Some women were even worried that their own movements could allow the device to expulse: “I realize that it can be placed far enough but …unless it is surgically placed and stitched into place, it can move …I mean what happens if you are walking down the street, you are running down the street and just, some people have been walking too fast and their babies have slid out [laughing]” (Jessica, Mixed, 33).

Of those women who were aware of appropriate IUD placement in the uterus, concern was voiced regarding potential harm to the uterus. “Or what if something happens and it is because of that and y'all have to go up in me to get it out and it would be too late because it already did so much damage inside of me? That is scary” (Aliyah, Black, 24). When prompted further, participants universally agreed that the ultimate harm they were worried about was the potential for infertility. Participants equated the uterus with their childbearing potential. One participant said, “I mean you have to take into consideration the risk factors like you know is this going to eventually make me infertile one day? Like is this going to potentially cause me to never be able to have kids if I choose to take it out or if something goes wrong” (Nicole, Hispanic, 26)? Another related it to a concern for infection, “So it kind of scares me placing something in there that could possibly get infected and especially into the uterus. If you lose your uterus, you can't have, I mean I have a bunch of children, but you can't have any more” (Jessica, Mixed, 33).

Related to positioning of the IUD in the body, multiple participants proposed the concept of “openness” of the uterus caused by an IUD. One participant responded, “So your uterus is open, like all the time” (Taylor, Black, 21)? In considering an IUD versus an implant, another said, “I don't know how far up it goes or just what happens… And the uterus is open, you have a direct shot there” (Jessica, Mixed, 33).

#### “Set and forget” concerns

When participants were asked why someone may not want an LARC “up in me,” adverse effects were commonly cited and were often related to the “set and forget” long-acting element of LARC methods. Participants expressed concerns about the safety of LARC devices that were explicitly regarding the safety of objects placed within their bodies, and not just general adverse effects of contraception. Women described their perception that an implanted device would cause problems over time: “And then, it is just scary, you about to place something in me that is going to stay in me for 5 years, like, and it's safe” (Aliyah, Black, 24)? The idea of an object sitting unmonitored in the body for years was unsettling.

Participants did not want to “forget” about their birth control, especially if is an object in their body. One woman expressed a comfort with depot medroxyprogesterone-acetate (DMPA) injections: “It makes me feel like I'm more protected than every 3 years [compared to the implant] because I'll forget about it…I'll forget that I even have it in me. Depo lets me remember” (Madison, Black, 16). A different participant also compared her lack of confidence in LARCs with her experience with DMPA, stating, “I don't know, it just, I guess getting the shot just you know the medicine is in there, and you know it is supposed to work” (Nicole, Hispanic, 26). One participant simply expressed skepticism at the duration of efficacy, “So something that is going to stay inside of me for years is going to stop pregnancy” (Lisa, Black, 48)?

Not only did participants not want to “forget” about their LARC devices, some wanted to be able to monitor them: “How do you know is it doing with it needs to do? … You know I don't have an X-ray machine. I just can't, you know at home, check on it every day in the mirror like I can do my make–up.” Even when counseled about the ability to check strings on an IUD, that participant did not feel reassured, “because I don't know anything about how long that string is supposed to be. I don't know what happens if you go up there and you can't find your string? Then you are going to freak out” (Nicole, Hispanic, 26).

#### Contraceptive devices versus other medical devices

We asked women to compare implantable contraceptives with other implantable medical devices, acknowledging that nonvaginally inserted devices are inherently different from IUDs (the previously determined focus of the phrase, “I don't want that up in me”). The major theme elicited was that contraception is medically elective compared with other medical devices, with subthemes including alternative options of contraception, IUDs not being needed for survival, and IUDs not easily monitored. In contrast, pacemakers are necessary for survival, breast implants are highly desired, and insulin pumps immediately demonstrate efficacy. One participant perceived less risk associated with a cosmetic procedure such as breast implants, and found them easier to monitor, “So you could like, you can tell that your implant is deflating or you can tell that your heart monitor is not working, it is not beeping it is not … but when your birth control is not working, you don't know… then you end up pregnant” (Madison, Black, 16). Another participant found the choice to be clear, “I feel like the diabetes pumps and all the other stuff is something you would need. I don't feel like you really need to have the birth control thing inserted in you. You can just take a pill. Or a shot” (Jordan, Black, 20). One participant elaborated,

So, with breast implants and things like that, all of those are completely cosmetic and that is something that you go in with the knowledge that it can't hurt you. Because it is cosmetic. With other things like a transplant or things like that, those are needed to sustain life. So, if you don't get them, you are going to die. 99% of the time, you are going to die or you are going to be walking around with 20 machines in your backpack…But with birth control because it is a preventative measure, I mean there are other ways to prevent having children. Like that is not the only option (Jessica, Mixed, 33).

#### Tracking device

Most of our interviews spontaneously turned into discussions about IUDs. However, we did have an unanticipated result about the etonogestrel implant. Two participants spontaneously expressed that an implanted contraceptive device could be a tracking device. One participant stated, “That is not normal for you to, it is like you have some tracking device in your arm” (Kayla, Black, 26). Another participant was responding to why a participant may have apprehensions regarding the implant, “It could be a tracking device” (Alexis, Black, 19).

## Discussion

Patient discomfort with the “foreign body” element of LARC methods has been demonstrated previously in the literature, but this theme has rarely been explored in depth. This study sought to investigate patients' concerns specifically related to the concept of an LARC as a foreign body and illuminates some of the worries and fears regarding this aspect of LARC devices.

One major element of “foreign body” discomfort is uncertainty about where the device resides in the body. In our sample, these worries were entirely about IUDs and not about implants. Concerns were about where the IUD is placed and whether it stays, which fit within the greater context of previously patient-identified negative effects of IUDs, including lack of information, side effects, insertion anxiety, infection risk, lack of control, partner's feeling it, possible pain and possible infertility.^13,21^ Certainly, if someone is unsure about where an IUD is and whether it moves around after placement, these fears are only reasonable. Meier et al. demonstrated that a prerequisite of effective shared decision-making is adequate information, which their sample perceived as primarily coming from provider/patient counseling.^22^ This finding of uncertainty regarding the interaction of patient anatomy and IUD placement offers a concrete target for providers to improve their counseling. We encourage providers to ensure that the terminology they use to explain where an IUD resides and how it functions in that space is understandable to the patient. Given the confusion about anatomic words, simple imagery illustrating these relationships cannot be undervalued.

Coupled with the likely alarm-inducing informed consent process, it should not be surprising that patients also expressed worries that any of these adverse effects may injure their uterus permanently and cause infertility. The Meier study also highlighted the importance of framing contraceptive discussions within the greater context of reproductive life goals and addressing possible desire for future fertility.^22^ In the context of our findings, not only is information about anatomy important, but also the reassurance that LARC devices are truly reversible and are unlikely to affect future childbearing.

Although prior studies have shown that patients and providers both agree that the “forgettable” nature of LARC can be an advantage,^10,12^ our study shows that some patients may consider it a disadvantage, specifically in the context of the LARC being considered a foreign body. Our participants explicitly expressed concerns that they would forget that they had an LARC device within them, and conversely appreciated methods that require more frequent follow-up (such as DMPA) to allow them to “remember” that they are protected from pregnancy, seen in the literature before.^9^

In addition, the “set and forget” advantages of LARCs inspired skepticism that a birth control method could be effective for that long. Participants wanted to be able to confirm that their devices would still be working but were frustrated by the available options for self-monitoring. Participant comments regarding leaving an LARC in their body for years at a time that may require monitoring or maintenance suggest a key difference appreciated by patients between an implantable medical device and a pharmaceutical contraceptive that has no physical presence.

In this study, we chose to probe the foreign body concept even further by asking participants to consider LARC devices in the context of other implanted medical devices. To our knowledge, this has not been explored before. We acknowledge that the comparison is artificial, but it did allow us to gain insight into how women perceive their contraception's role in their own wellness. Participants were consistent in stating that the primary difference between contraception and other medical devices is that there are alternative options available for birth control and that contraception is not life-sustaining. They also highlighted that in comparison, it is difficult to monitor the continued efficacy and safety of these implanted devices. In this perspective, LARC devices are not medically necessary and may not be worth the perceived risks.

Remarkably, two women also spontaneously expressed concerns that the implants could be “tracking devices,” reminding providers that patients have legitimate historical and contemporary reason to be skeptical of the medical profession. Providers have been shown to preferentially recommend LARC devices to women of color and poor women.^23^ Patients are aware of provider biases and historical instances of reproductive coercion, such as forced sterilization in the name of Eugenics and coercive Norplant for welfare recipients.^24,25^ Burns et al. showed in their survey of women exploring LARC awareness and interest that 3% and 9% of surveyed women were not interested in the IUD and implant because of perceived misuse of the method against their communities.^4^ The spontaneous declaration that a foreign body could be implanted for the purpose of secretive surveillance, especially coming from two young black women, is a reminder to us all that the legacy of prior injustices persists within marginalized communities today.

Applying the lens of reproductive justice to the findings of this study highlights the importance of women's understanding, engagement, comfort, and consent with their contraceptive choices. Ambiguity regarding device positioning is out of the patient's control and forces them to appeal to a medical provider to assess or solve a positioning problem. The threat of infertility caused by LARC damage can rob a patient of her reproductive autonomy. The “set and forget” element can also be understood as removing agency from the patient's management of her own reproduction. Understanding that issues of reproductive coercion are present in all matters of contraceptive choice and not just overt matters of contraceptive implants as tracking devices is imperative to us all as providers.

By design, our use of a purely qualitative descriptive approach does not seek to claim generalizability. Alternate themes may have emerged had we sampled from a different region or included women with different characteristics such as higher socioeconomic circumstances or higher levels of educational attainment. Likewise, sampling from a younger population such as adolescents may yield different themes. Although our sample was not stratified by prior experience with LARC use, our participants were all familiar with LARCs and were readily able to offer their perspectives. Our sample was mostly multiparous and mostly identified as black, which are representative of our clinic population. Recent National Survey of Family Growth data suggest that LARC use is similar between women of different race/ethnicity, but does differ by parity,^2^ and thus, a sample with more nulliparous women may reveal different considerations on this topic. A strength of this study is that thematic saturation was reached at about the 20th interview, with remaining interviews confirming no new content or codes. We were also able to gather rich perspectives on how women conceptualize foreign body contraceptives. Some weaknesses include that the participants self-selected in their willingness to participate in a study about contraception and thus may represent those with stronger opinions about contraceptive methods.

## Conclusion

This study offers new insights into participant decision-making about LARC devices. It offers clear targets for patient education regarding accurate anatomy, to offer reassurance that most IUDs do not move and why and how LARC devices can be effective for so long. Our findings that participants view contraception as being elective compared with other medical interventions and our unexpected finding that implants were perceived as tracking devices also suggest that perhaps the most crucial part of contraceptive counseling is practicing within the scope of a reproductive justice framework.^26^

Our findings provide an in-depth understanding regarding the perceptions and beliefs regarding foreign body contraceptive devices. These findings suggest the need to find new strategies and approaches to address existing distrust of medical institutions, particularly regarding reproductive health services, and better elicit and engage with women's priorities, values, and beliefs. In this manner, we hope this understanding will inform contraceptive counseling approaches that incorporate more patient-centered approaches in exploring woman's concerns about safety and efficacy as well as her own reproductive goals and needs. We hope to move past the “LARC first” and tiered contraceptive counseling strategies to counseling approaches that, as so well put by Gomez et al., “put the priorities, needs and preferences of individual women—not the promotion of specific technologies—first.”^27^

## Abbreviations Used

DMPA: depot medroxyprogesterone-acetate
IUD: intrauterine device
LARC: long-acting reversible contraception
